# Mapping the use of mental imagery for stress management in athletes: a scoping review

**DOI:** 10.3389/fpsyg.2026.1931753

**Published:** 2026-09-23

**Authors:** Luxia Zhao, Yan Wang, Nurwina Anuar

**Affiliations:** Faculty of Education, Universiti Kebangsaan Malaysia, Bangi, Malaysia

**Keywords:** athletes, injury rehabilitation, mental imagery, psychological skills, scoping review, sport performance, stress management

## Abstract

**Introduction:**

Mental imagery is widely used in sport, yet its specific role in helping athletes manage psychological stress has not been systematically mapped, and the term is applied to distinct constructs that are frequently conflated. This scoping review charted the extent, range and nature of the evidence on mental imagery for stress management in athletes, including performance, psychological fatigue and injury-related contexts.

**Methods:**

Web of Science, Scopus, PubMed and EBSCOhost were searched in January 2024, with an updated search using the same strategy and databases on 27 July 2026. Eligibility was defined using a Population-Concept-Context framework, and reporting followed PRISMA-ScR. After duplicate removal, 1,004 records were screened by two independent reviewers. Retained sources were classified by their role in the review, and imagery was coded on four dimensions: imagery ability, imagery content and function, imagery use, and intervention format. Data were synthesised descriptively and narratively; no meta-analysis or formal quality appraisal was undertaken, consistent with the purpose of a scoping review.

**Results:**

Thirty-nine core empirical studies met both operational gates and were charted, 20 from the original search and 19 from the update; a further 94 sources were retained for narrative or contextual framing. Nineteen (49%) of the charted studies were published in 2023 or later. Of the 39 charted studies, 24 (62%) examined psychological mechanisms, 21 (54%) addressed performance outcomes and psychological fatigue, 13 (33%) examined individual and sport-specific variation, and 7 (18%) concerned injury prevention and rehabilitation. Imagery was most often linked to stress management through appraisal- and emotion-related processes, was commonly embedded within multicomponent psychological-skills programmes, and varied by athlete and sport context. Null and inconclusive findings were also identified, including divergent mediation results and unmet feasibility criteria.

**Discussion:**

Associations involving imagery ability or imagery use do not by themselves establish intervention efficacy, and outcomes from multicomponent programmes cannot usually be attributed to imagery alone. The evidence map supports clearer construct definitions and component-controlled, longitudinal research rather than causal conclusions about a uniform imagery intervention.

## 1 Introduction

Psychological stress is an inherent feature of competitive sport, arising from performance demands, evaluative pressure, intensive training, and the constant risk of injury (Hardy, 1992; Williams and Andersen, 1998; Rumbold et al., 2012). When inadequately managed, such stress can disrupt athletes’ emotional regulation and coping responses, with potential consequences for both athletic performance and psychological well-being (Gould et al., 1993; Rumbold et al., 2012). Consequently, identifying psychological strategies that support stress management has become an important focus of contemporary sport psychology. Among these strategies, mental imagery offers a particularly relevant lens for understanding how athletes mentally prepare for and cope with sport-related stress.

Mental imagery involves the internal simulation or recreation of sport-related experiences without corresponding physical action. It is widely regarded as a core psychological skill within sport psychology, allowing athletes to mentally rehearse movements, competitive situations, and coping responses in preparation for performance and other sport-related demands (Anshel, 2005; Johnson and Gilbert, 2004; Ziv and Lidor, 2013). Accordingly, mental imagery has been incorporated into a broad range of sporting contexts, including skills training and competition preparation, providing a flexible psychological approach across different stages of athletic participation (Coelho et al., 2012; Van Raalte et al., 2019; Ziv and Lidor, 2013).

The proposed effects of imagery are underpinned by several theoretical accounts. Bio-informational theory holds that imagery activates stimulus, response, and meaning propositions, such that rehearsing the physiological and emotional response elements of a scene can shape subsequent arousal and appraisal (Lang, 1979). The functional-equivalence account proposes that imagery and actual perception or movement share overlapping neural representations, providing a basis for imagery to influence motor readiness and emotional processing (Jeannerod, 1994). Applied frameworks such as PETTLEP translate these principles into practice by emphasising the physical, environmental, task, timing, learning, emotion, and perspective components that make imagery more vivid and functionally equivalent to performance (Holmes and Collins, 2001). Together, these accounts suggest that imagery is not merely mental picturing but a structured simulation capable of engaging the appraisal and emotion-regulation processes central to stress management, which motivates examining how it has been applied for that purpose in athletes.

To prevent distinct forms of evidence from being treated as interchangeable, this review distinguishes four dimensions of imagery. Imagery ability is the perceived capacity to generate and control images, including ease, vividness, and controllability (Williams and Cumming, 2011; Lee et al., 2025). Imagery content and function describe what is represented and the intended role of that representation, including skill, strategy, goal, affect, and mastery functions (Cumming and Williams, 2013; Lee and Uhm, 2026). Imagery use describes how frequently, when, where, and for what purpose athletes deliberately image. Intervention format describes how imagery is delivered, for example as a standalone script, guided or PETTLEP-based practice, or one component of a multicomponent programme. These dimensions are related but not interchangeable. An association involving imagery ability or use does not demonstrate that imagery training is effective, and an outcome following a multicomponent programme cannot automatically be attributed to imagery.

A growing body of research has examined the application of mental imagery in relation to stress-related psychological outcomes among athletes (Rumbold et al., 2012; Coelho et al., 2012). Existing studies have reported that mental imagery, frequently incorporated into broader psychological skills and stress-management interventions, is associated with more adaptive anxiety and stress responses in athletes (Williams et al., 2017; Quinton et al., 2019; Mace and Carroll, 1989; Vesković et al., 2019). In addition, mental imagery has been associated with greater confidence and enhanced psychological readiness for performance, highlighting its broader role in supporting athletes’ psychological functioning beyond anxiety regulation (Williams et al., 2021; Westlund et al., 2012). At the same time, the existing evidence varies considerably in terms of imagery approaches, participant characteristics, and outcome measures, with mental imagery being examined both as a standalone strategy and as part of broader psychological interventions (Williams et al., 2017; Westlund et al., 2012; Mace and Carroll, 1989).

Existing reviews have addressed adjacent but distinct questions. Syntheses of psychological interventions and stress-management strategies in sport have considered imagery as one technique among many rather than as their focus (Rumbold et al., 2012); reviews of the stress–injury relationship and of psychological approaches to injury have examined imagery within rehabilitation and prevention (Schwab Reese et al., 2012); and broader psychological-skills-training literatures treat imagery as one skill within multicomponent programmes. What has not been established is a map of how mental imagery specifically has been used for stress management in athletes, spanning its mechanisms, its role in performance and psychological fatigue, individual and sport-specific variation, and its place in injury prevention and rehabilitation, and how that evidence is distributed across designs, populations, and outcomes. Because this question concerns the extent, range, and structure of a dispersed and heterogeneous literature rather than the pooled efficacy of a defined intervention, a scoping review is the appropriate approach (Arksey and O’Malley, 2005; Levac et al., 2010). Mapping the field in this way clarifies what is known, identifies gaps, and establishes whether the evidence is yet suited to more constrained forms of synthesis such as a systematic review or meta-analysis.

Accordingly, this scoping review aimed to map the existing literature on the use of mental imagery for stress management among athletes. Specifically, it sought to characterise how mental imagery has been applied for stress management across athlete populations and sporting contexts, including performance and injury-related contexts such as prevention and rehabilitation. It further sought to identify the stress-, performance-, and injury-related outcomes examined, individual and sport-specific variations in its use, and gaps that remain for future research. By bringing this evidence together, the review seeks to provide researchers and practitioners with an integrated overview intended to guide further investigation and evidence-informed psychological practice in sport.

## 2 Materials and methods

### 2.1 Study design and protocol

This study was conducted as a scoping review to map the extent and nature of available evidence on the use of mental imagery for stress management among athletes. A scoping review approach was considered appropriate because the review aimed to characterise how the topic has been studied, identify key themes and knowledge gaps, and clarify the range of contexts in which mental imagery has been applied, rather than evaluate the efficacy of specific interventions. The review was guided by the methodological framework proposed by Arksey and O’Malley (2005) and further refined by Levac et al. (2010). Reporting was informed by the Preferred Reporting Items for Systematic Reviews and Meta-Analyses extension for Scoping Reviews (PRISMA-ScR; Tricco et al., 2018), and the selection process is reported using the PRISMA 2020 flow diagram template (Page et al., 2021). No formal review protocol was registered for this scoping review; however, the research questions, eligibility criteria, and data-charting framework were established in advance to guide the review process.

### 2.2 Search strategy and information sources

A structured literature search was conducted across four electronic databases: Web of Science, Scopus, PubMed, and EBSCOhost. The initial search was conducted in January 2024, and an updated search using the same strategy was conducted in July 2026. The search strategy combined terms related to mental imagery, athletes or sport participation, and stress management or related psychological outcomes. Search terms included combinations of keywords such as “mental imagery,” “imagery,” “visualisation,” “visualization,” “athlete,” “sport,” “stress,” “anxiety,” “coping,” “performance pressure,” “injury prevention,” and “rehabilitation.” The initial search had no year limit. No language restriction was applied at the search stage; records published in languages other than English were excluded during screening in accordance with the eligibility criteria. The search concepts, keyword combinations, and Boolean logic are presented in Supplementary Table S1.

The initial search identified 563 records: 101 from Web of Science, 24 from Scopus, 416 from PubMed, and 22 from EBSCOhost. Thirty-eight duplicates were removed, leaving 525 records for title and abstract screening. The updated search was conducted on 27 July 2026 using the same strategy and databases, with coverage from January 2023 to July 2026. It identified 941 records: 254 from Web of Science, 347 from Scopus, 121 from PubMed, and 219 from EBSCOhost. After deduplication, including removal of 175 records duplicated within the EBSCOhost export, 462 duplicate records were removed from the updated set, leaving 479 unique records. Across both search waves, 1,504 records were identified, 500 duplicates were removed, and 1,004 records underwent title and abstract screening. The search concepts, keyword combinations, Boolean structure, database-specific yields, and dated deduplication audit are reported in Supplementary Table S1.

### 2.3 Eligibility criteria

Eligibility criteria were defined in relation to the population, concept, context, and source type relevant to the review objectives. Sources were eligible if they addressed athletes, sport participants, or sport-related performance, training, injury-prevention, or rehabilitation contexts. The central concept of interest was the use of mental imagery, including mental rehearsal, imagery-based psychological skills, or psychological skills interventions in which imagery was a clearly identifiable component. Eligible contexts included stress management, anxiety, coping, performance pressure, injury prevention, and rehabilitation in sport. Eligibility is framed with the Population–Concept–Context structure recommended for scoping reviews rather than with PICOS, because the review maps the extent and nature of a heterogeneous literature rather than estimating an effect: no comparator was required for eligibility, and requiring one would have excluded the descriptive, correlational, and single-group designs that constitute much of the available evidence (Tricco et al., 2018).

A broad range of peer-reviewed sources was considered eligible, including empirical studies of any design, as well as relevant reviews and conceptual or applied sources, provided that they addressed mental imagery or imagery-based psychological skills in relation to sport-related stress management or associated performance and injury-related contexts. Sources were excluded if they were not related to sport or athletic contexts, did not address mental imagery or an imagery-based psychological skill, focused solely on medical, biological, or physiological outcomes without a relevant psychological component, were not related to stress management or associated psychological outcomes, were duplicates, were unavailable in full text, or were published in languages other than English.

Because the boundary of the imagery construct determines which sources enter the charted synthesis, the operational rules applied at full-text assessment are stated explicitly here. Two gates had to be satisfied for a study to enter the charted evidence synthesis as a core empirical study. First, imagery had to be operationally distinct: it had to be manipulated as an identifiable intervention component, measured with an imagery-specific instrument, or analysed as a separate variable. Studies in which imagery appeared only as one unanalysed element of a multicomponent psychological-skills package, or only as one subscale among many parallel subscales of a general mental-skills inventory, did not satisfy this gate. Second, the study had to report at least one eligible psychological outcome. Studies whose only outcomes were physiological indices, motor-performance accuracy, or cognitive-task scores did not satisfy this gate, and neither did studies in which imagery ability was itself the sole dependent variable with no psychological outcome measured alongside it. These two rules were applied to the sources retained in the original review as well as to the updated search. Three studies charted as core empirical in the original submission were reclassified as supporting sources: two because imagery was only one subscale in a general mental-skills inventory, and Slimani et al. (2017) because it reported no eligible psychological outcome. Sources useful for theoretical, methodological, or contextual framing but not meeting both gates were retained as supporting or background references where relevant and were not entered into the charting table. The operational decisions and exclusion codes were recorded in the screening log.

### 2.4 Study selection and data charting

Following each search, database exports were consolidated and duplicates were removed before screening. ZL and WY independently screened the titles and abstracts of all 1,004 de-duplicated records against the prespecified eligibility criteria. Reports judged potentially eligible by either reviewer proceeded to retrieval. Across both search waves, 196 reports were sought, 18 could not be retrieved, and 178 were assessed for eligibility.

At the full-text stage, each reviewer applied the two operational gates described in Section 2.3 to the reports retained through their own independent screen. WY retained 71 reports after full-text assessment. Comparison of the reviewers’ core-study classifications identified five discrepant reports. Each discrepancy was discussed against the operational gate definitions, resulting in two reports being included in the final core empirical set. Final inclusion, exclusion, and source-role decisions were agreed by consensus and recorded in the screening log.

Of the 178 reports assessed at full text, 45 were excluded: 29 because no relevant imagery–stress relationship was reported, two because they duplicated an already included report, and 14 because they did not satisfy the imagery-centrality or psychological-outcome gates. Reports initially unavailable through institutional channels but subsequently supplied by their authors were included in full-text assessment. ZL conducted the primary data charting. The complete selection process is summarised in Figure 1, and search-wave details are reported in Supplementary Table S1.

**Figure 1:**
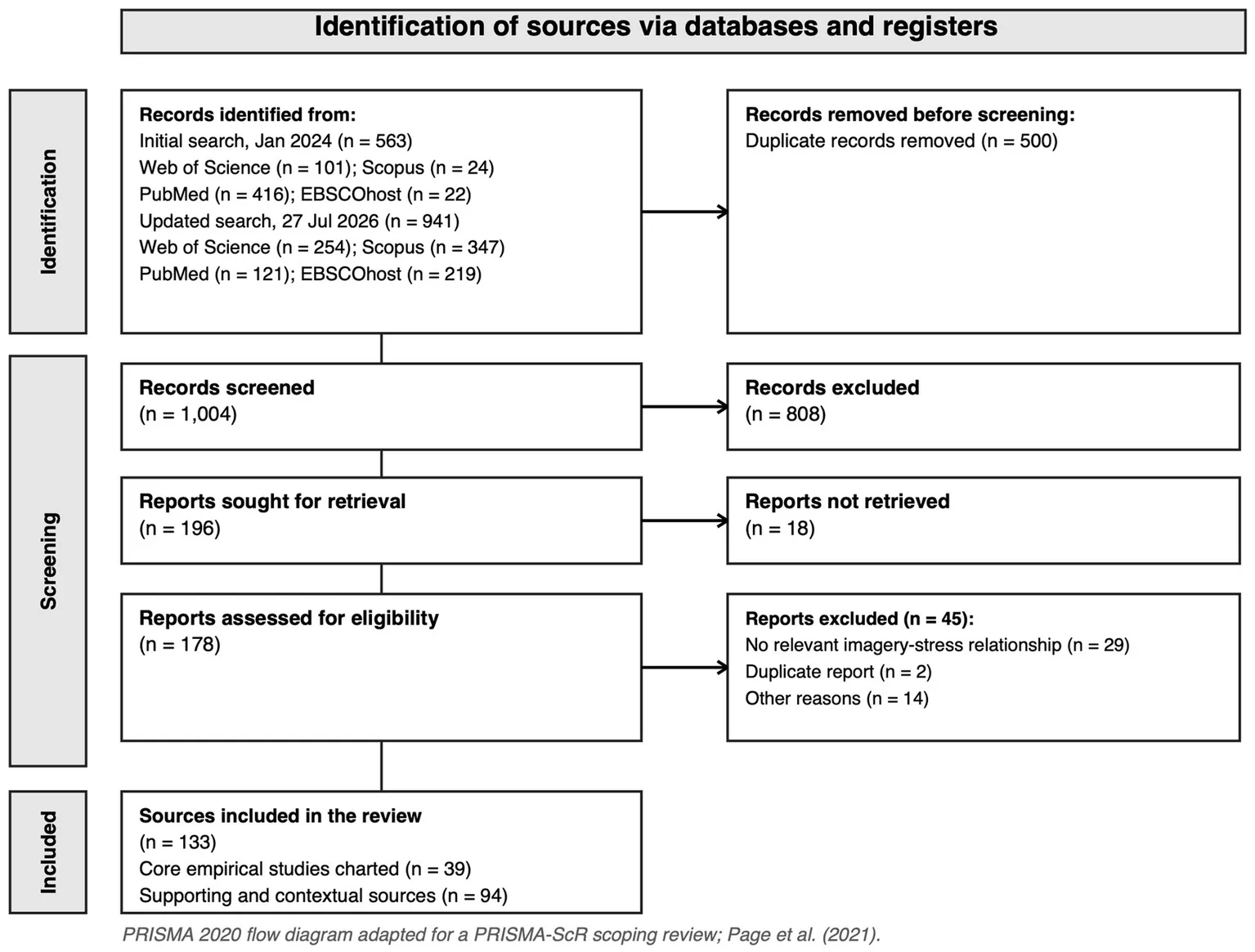
PRISMA flow diagram of source identification, screening, and eligibility assessment across both search waves, prepared using the PRISMA 2020 flow diagram template. Retained sources are separated into the core empirical studies charted for synthesis and the supporting and background sources retained for narrative or contextual framing. Under “Other reasons,” the 14 reports excluded at full text in the updated search wave comprised records that did not meet the imagery-centrality or psychological-outcome gates on full-text assessment. Figure is adapted from Page et al. (2021). Creative Commons Attribution 4.0 International (CC BY 4.0).

During data charting, retained sources were classified according to their role in the review. Thirty-nine core empirical studies satisfied both gates described in Section 2.3 and were entered into the charted evidence synthesis: 20 from the original search and 19 from the updated search. These studies are reported in Supplementary Table S2. A further 94 review, conceptual, applied, adjacent empirical, and background sources were retained for narrative or contextual use but were not entered into study-level charting. These supporting sources did not contribute to the core evidence counts. Several sources addressed more than one thematic area.

Data were charted using a structured extraction form that captured author and year, source type, study design, participant and sport context, psychological outcomes, key findings, and reported limitations. Imagery was additionally coded using the four-dimensional framework shown in Figure 2: imagery ability; imagery content and function; imagery use; and intervention format. A study could contribute to more than one dimension, but the dimensions were not collapsed. Ability and use studies were interpreted as associational unless an experimental manipulation supported a stronger inference. Standalone and embedded interventions were coded separately, and outcomes from multicomponent programmes were not treated as imagery-specific effects. The data-charting process was iterative, with categories refined as familiarity with the sources increased.

**Figure 2:**
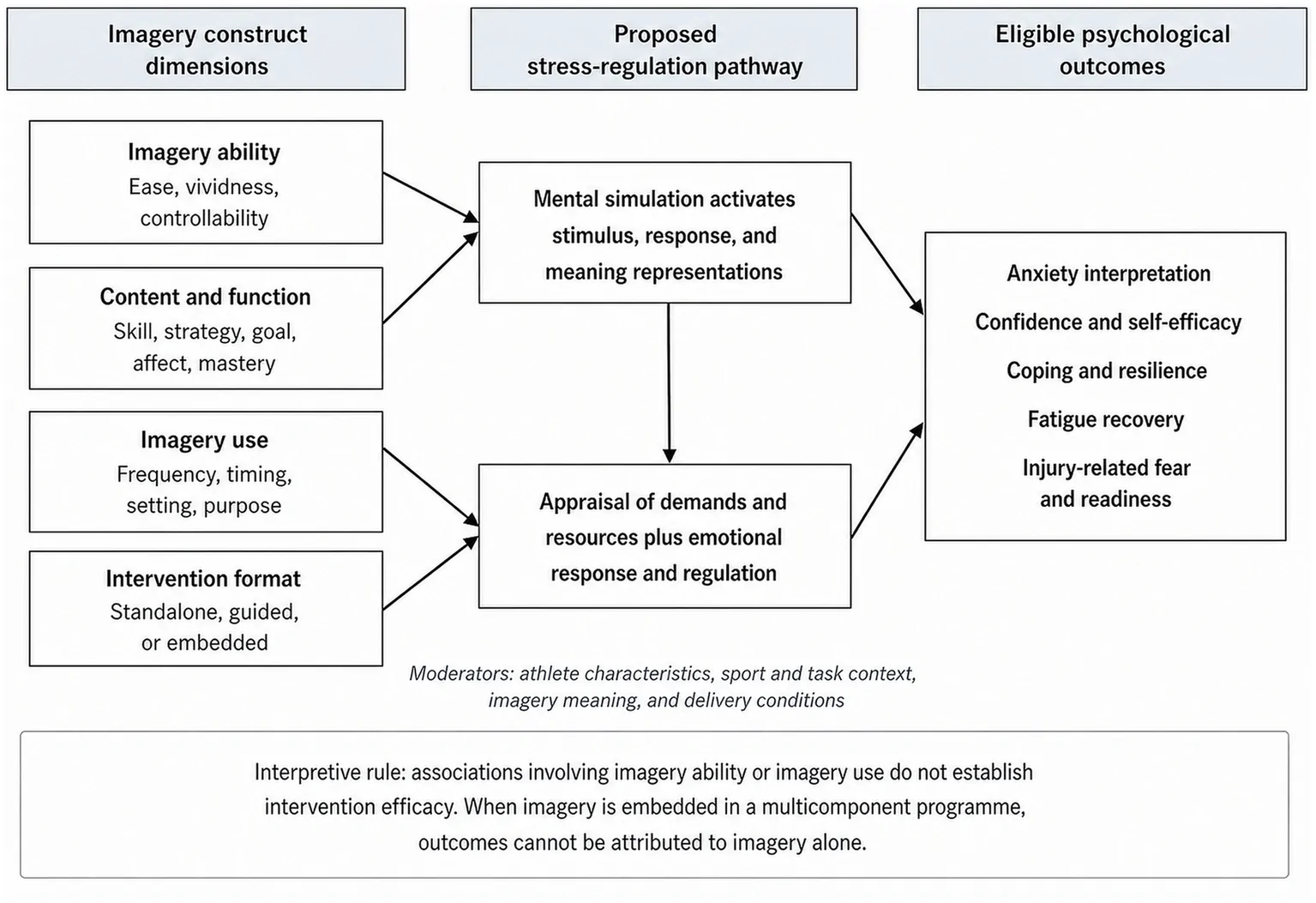
Analytical framework distinguishing imagery ability, imagery content and function, imagery use, and intervention format, and showing their proposed relationships with stress-related psychological outcomes. The framework draws on bio-informational theory, functional equivalence, PETTLEP, and the revised applied model of deliberate imagery use (Lang, 1979; Jeannerod, 1994; Holmes and Collins, 2001; Cumming and Williams, 2013).

### 2.5 Data synthesis and methodological appraisal statement

Data were synthesised descriptively and narratively in accordance with the objectives of a scoping review. Descriptive summaries characterised source type, design, athlete population, sport context, the four imagery dimensions, and outcomes. The narrative synthesis then organised the charted evidence around stress-regulation mechanisms, performance and psychological fatigue, individual and sport-specific variation, injury prevention and rehabilitation, and remaining gaps. The charted evidence spanned 1989 to 2026.

Given the heterogeneity of source types, study designs, imagery approaches, and outcome measures, no meta-analysis or pooled effect-size estimation was conducted. In line with the purpose of a scoping review, the synthesis aimed to map the breadth and nature of the available evidence rather than evaluate the efficacy of specific interventions.

A formal methodological quality appraisal was not undertaken, as the review was designed to map the field rather than assess risk of bias or determine intervention effectiveness. To support transparency, sources were classified according to their role in the review, and distinctions were maintained between core empirical imagery studies, review/conceptual/applied imagery-related sources, and sources retained only for contextual or background framing.

## 3 Results

### 3.1 Characteristics of the charted evidence

Across both search waves, 1,004 de-duplicated records were screened and 133 sources were retained for synthesis. Of these, 39 met both operational gates and were charted as core empirical studies, comprising 20 from the original search and 19 from the updated search. The remaining 94 sources were retained for supporting narrative or contextual use. Figure 1 presents the full selection flow, Table 1 provides the condensed evidence map, and Supplementary Table S2 provides the study-level charting record.

**Table 1:** Condensed evidence map of charted studies (***n*** = 39).

Re**f**	Author (year)	Sample/sport	Design/source type	Imagery approach	Outcome(s) measured	Theme(s)
1	Mace and Carroll (1989)	Gymnasts	Experimental / intervention (SIT)	Imagery within stress-inoculation training	Self-report/observer stress / HR / performance	Mechanisms (multimodal); Performance
2	Gould et al. (1992)	Olympic wrestlers	Qualitative / interview	Mental preparation incl. Mental rehearsal/visualization	Mental preparation / precompetitive cognition / affect	Individual/sport-specific; Performance
3	Gould et al. (1993)	National-champion figure skaters	Qualitative / interview	Imagery among emotion-regulation & cognitive-control strategies	Coping / emotion regulation / performance under stress	Individual/sport-specific; Performance
4	Carter and Kelly (1997)	Intramural basketball players	Experimental	Confidence vs. contradictory imagery	Anxiety / confidence (by reactance)	Individual (reactance); Mechanisms
5	Ryska (1998)	Recreational tennis players	Cross-sectional survey	Mental imagery (four tennis-specific referents); merged with relaxation for analysis owing to collinearity (r = 0.81)	Precompetitive anxiety / strategy use	Individual; Performance
6	Johnson (2000)	Long-term-injured competitive athletes	Intervention (rehab)	Guided imagery	Mood / psychological recovery	Injury (recovery)
7	Wang et al. (2003)	Athletes (choking under pressure)	Intervention / applied	Psychological intervention incl. Imagery	Choking / pressure / imagery practice	Performance; Individual (age)
8	Davis and Sime (2005)	Injured athlete(s) (case-based)	Case study	Imagery + EEG biofeedback	Confidence / performance post-recovery	Injury (recovery)
9	Mellalieu et al. (2009)	Male rugby union players	Single-case intervention	Motivational general-arousal (MG-A) imagery	Pre-performance symptoms / anxiety interpretation	Performance
10	Coelho et al. (2012)	Elite tennis players	Intervention (pre–post)	Multimodal imagery intervention	Pre-comp anxiety↓ / stress↓ / confidence↑	Mechanisms (multimodal); Performance
11	Westlund et al. (2012)	Athletes	Cross-sectional / correlational	Cognitive general (CG) imagery	Confidence (CG imagery association)	Individual (confidence); Mechanisms
12	Olmedilla-Zafra et al. (2017)	Young football players	Intervention (prevention)	Imagery within a stress-management programme	Injury incidence	Injury (prevention)
13	Williams et al. (2017)	Lab acute-stress sample (mixed sex)	Experimental (imagery scripts)	Challenge vs. threat imagery scripts	Anxiety / cardiovascular (HR) / appraisal	Mechanisms; Individual (gender)
14	Olmedilla et al. (2019)	Youth soccer players	Intervention (prevention)	Imagery within a psychological programme	Stress control / injury incidence	Injury (prevention); Performance
15	Quinton et al. (2019)	Athletes (acute stress task)	Experimental (imagery scripts)	Mastery imagery (ability/scripts)	Stress responses / emotion regulation / appraisal	Mechanisms
16	Vesković et al. (2019)	Top karate athletes	Intervention (PST)	Imagery within psychological-skills training	Competitive anxiety	Performance/anxiety; Sport-specific (combat)
17	Loch et al. (2020)	Mentally fatigued participants	Experimental	Imagery + breathing as recovery strategy	Psychological fatigue / attention / cognitive performance	Performance (fatigue & recovery)
18	Schlatter et al. (2020)	Athletes (acute stress; pilot)	Experimental (pilot)	Implicit vs. explicit motor imagery	Implicit vs. explicit motor imagery under stress	Mechanisms (motor imagery); Sport-specific
19	Williams et al. (2021)	Athletes (psychological stress)	Experimental	Mastery imagery ability	Positive anxiety / performance / confidence	Mechanisms; Performance; Individual
20	Isorna-Folgar et al. (2022)	Junior national-team rowers	Intervention (CBT)	Imagery within a CBT programme	Motivation / concentration / cohesion / performance	Performance
21	Barbosa et al. (2023)	519 adolescent athletes (14–19 yr)	Cross-sectional / SEM	Sport Imagery Questionnaire (SIQ) in a mediation model	Sport confidence; competitive performance	Mechanisms; Performance
22	Budnik-Przybylska et al. (2023)	30 participants, 14–42 yr., laboratory	Laboratory correlational psychophysiology	Imagery Skills Questionnaire; Sport Anxiety Scale; skin conductance level (SCL/EDA)	Physiological arousal (SCL) during imagery; sport anxiety; neuroticism as moderators	Mechanisms; Individual/sport-specific
23	Lu and Xu (2023)	120 basketball players, China	Intervention (pre-post, no control)	PETTLEP imagery	Self-report psychological outcomes	Mechanisms; Performance
24	Sharma and Prasad (2023)	50 male state-level table tennis players, India (25 VMBR / 25 control), 17–23 yr	Randomised controlled trial	Visuo-motor behaviour rehearsal (imagery + relaxation)	Anxiety dimensions (somatic, worry, concentration disruption); mental toughness (confidence and consistency subscales non-significant)	Mechanisms; Multimodal
25	Ali and Kazmi (2024)	58 student-athletes, Pakistan	Cross-sectional / mediation	Healing imagery (measured)	Competitive sport anxiety; achievement goals	Mechanisms
26	Cederström et al. (2024)	42 people rehabilitating traumatic knee injury (MOTIFS vs. care-as-usual, 12 weeks)	Randomised controlled trial (adaptive; interim analysis)	Integrated motor imagery plus physical training	Psychological readiness to return to sport	Injury (recovery)
27	Emirzeoglu et al. (2024)	53 elite athletes	Cross-sectional / correlational	MIQ-3 internal, external and kinaesthetic imagery ability	Body awareness; athletic psychological skills subscales (largely null; one negative association involving worry)	Mechanisms (null findings); Individual/sport-specific
28	Khongrassame et al. (2024)	24 collegiate futsal players, Thailand	Intervention (8-week PETTLEP), one-group pre-post, no control group	PETTLEP imagery, 8 weeks	Shooting skill; competitive anxiety; self-confidence	Mechanisms; Performance
29	McAlister et al. (2024)	21 female Division I volleyball players, USA	Pilot feasibility RCT	Imagery vs. mindfulness (separate arms)	CSAI-2R anxiety and self-confidence; feasibility	Mechanisms; Performance
30	Plakoutsis et al. (2024)	58 professional footballers with Grade II ankle sprain (29 motor imagery / 29 placebo), Greece	RCT	Motor imagery added to rehabilitation	Static and dynamic balance; heart rate; fear of re-injury (no between-group difference on fear of re-injury)	Injury (recovery)
31	D'Agostino and Munroe-Chandler (2025)	243 student-athletes, Canada and USA	Cross-sectional / SEM	SIQ five imagery functions	Psychological resilience (CD-RISC); sport confidence as mediator	Mechanisms
32	Demir et al. (2025)	407 elite athletes (143 female / 264 male), Türkiye, 19–26 yr	Cross-sectional / SEM	Sports Imagery Inventory (SII); Mental Toughness Scale; ESS anxiety subscale	Mental toughness; anxiety (SEM paths; athletic performance not measured)	Mechanisms; Performance
33	Di Corrado et al. (2025)	110 karate, taekwondo and judo athletes, Italy	Cross-sectional / mediation	VVIQ, VMIQ-2 and a mental image transformation task	Perceived stress; general self-efficacy as mediator	Mechanisms
34	Norouzi et al. (2025)	*n* = 36 elite female handball players, post-COVID-19 motor function reduction	Randomised controlled trial (three arms), pre-test / post-test / 12-week follow-up	Functional imagery training (FIT, imagery + motivational interviewing) vs. motor imagery; 12 sessions over 4 weeks, 3 × 30 min per week	DERS (emotion dysregulation); BAI (anxiety); PSS (perceived stress); NSMT reaction time (DERS, BAI and PSS declined in all three groups; the imagery advantage was confined to reaction time)	Anxiety and stress regulation; emotion regulation
35	Olds (2025)	*n* = 1 male athlete aged 23, recurrent shoulder instability after repeated stabilisation surgery, New Zealand	Single-case report	Graded exposure to fear-eliciting images used as a first-person motor imagery task, adjunct to usual physiotherapy	Kinesiophobia (TSK-11); per-image fear rating; shoulder strength and range of motion	Injury (recovery); mechanisms
36	Pratama Sudrajat et al. (2025)	5 female pistol shooters, Indonesian Army	Intervention (one-group pre-post)	Mental imagery, 5 sessions	CSAI-2R competitive anxiety	Mechanisms; Performance
37	Di Corrado et al. (2026)	120 male adolescent rugby players, Italy (62 experimental / 58 control), mean age 16.9 yr	Randomised controlled trial	Personalised mental imagery training	Anger expression; resilience	Mechanisms; Individual/sport-specific
38	Oldfield et al. (2026)	24 footballers	Within-subject experimental	Imagery measured after each crowd-noise condition	Imagery, self-talk, relaxation and emotional control; penalty performance (no psychological skill, including imagery, moderated the crowd-noise effect)	Mechanisms; Performance
39	Pakulanon and Petviset (2025)	30 university athletes, Thailand (badminton, athletics, football and swimming), 18–25 yr	Three-arm randomised controlled trial (*n* = 10 per arm), 8 weeks	MG-A/MG-M guided imagery vs. mindfulness meditation vs. relaxation-brochure control	CSAI-2R cognitive and somatic anxiety and self-confidence; HRV (supporting physiological outcome)	Performance/anxiety (null findings)

The full charting record, including source roles and reporting caveats, is provided in Supplementary Table S2.

The charted studies were published between 1989 and 2026. The updated search substantially shifted the temporal profile of the evidence: 19 (49%) of the charted studies were published in 2023 or later, so that almost half of the current evidence base postdates the original search. The charted studies addressed several overlapping thematic areas. Multiple coding was permitted, so the 39 studies generated 65 theme assignments and the percentages below do not sum to 100%: 24 (62%) examined psychological mechanisms through which imagery relates to stress management (Section 3.2); 21 (54%) addressed imagery in relation to performance outcomes and psychological fatigue (Section 3.3); 13 (33%) examined individual and sport-specific variation in imagery use (Section 3.4); and 7 (18%) concerned injury prevention and rehabilitation (Section 3.5). Because many studies spanned more than one area, these categories are not mutually exclusive. All charted studies sampled athletes or sport participants; sources addressing sport practitioners such as coaches, athletic trainers, or physical therapists were retained as supporting evidence rather than charted, because the athlete population criterion was not met.

A range of designs was represented. Classified by whether imagery was delivered or experimentally manipulated rather than only measured, 28 studies delivered or manipulated imagery, 9 measured imagery as a construct without manipulating it, and 2 were qualitative interview studies. Within the 28 studies in which imagery was delivered or manipulated, 7 were randomised controlled trials, 15 were non-randomised group interventions or laboratory manipulations, 3 were single-group pre-post designs without a control condition, and 3 were single-case reports. Randomised designs were concentrated in the more recent literature: all seven randomised controlled trials were published in 2023 or later. Several of the more recent intervention studies were explicitly designated pilot or feasibility work, and several had very small samples, in one case five participants with no control group. This distribution reflects the characteristic breadth of a scoping review, which maps evidence across design types rather than restricting synthesis to a single design, but it also means that the balance of evidence supports description of how imagery is used more strongly than estimation of its isolated effect.

The sampled populations were heterogeneous and spanned recreational, collegiate, elite, and Olympic-level athletes, alongside a small number of laboratory acute-stress and mentally fatigued samples. Sport contexts included combat sports (wrestling, judo, karate, taekwondo, and kickboxing), team and ball sports (soccer, futsal, volleyball, basketball, and rugby), racket sports (tennis and table tennis), aesthetic sports (figure skating and gymnastics), shooting, and endurance and sprint running, as well as mixed-sport and injured-athlete samples. The updated search broadened the geographical spread of the charted evidence: whereas the originally charted studies were concentrated in European and North American settings, the studies added by the updated search include samples from Thailand, Pakistan, India, Indonesia, Iran, Italy, Tunisia, Türkiye, Greece, and Sweden. Because geographic setting was not consistently charted or verified across the original source records, it is not treated as a primary mapping variable in the evidence map, but the addition of these samples partially addresses the under-representation of non-Western populations noted in the original synthesis.

Sport-type differences also appear in sources retained for supporting rather than charting purposes. Warfield et al. (2023) reported sport-type differences in relaxation and imagery preferences among NCAA Division I athletes but was retained as supporting evidence because imagery was not analysed against a stress-related psychological outcome.

The four-dimensional framework clarified why the charted evidence should not be treated as one homogeneous intervention literature. Studies of imagery ability assessed ease, vividness, controllability, or modality-specific capacity. Studies of content and function examined what athletes imagined and why, including challenge, threat, mastery, skill, strategy, goal, affect, or rehabilitation content. Studies of imagery use measured frequency, timing, setting, or purpose. Intervention studies varied in format, ranging from standalone scripts, guided imagery, motor imagery, and PETTLEP practice to imagery embedded within relaxation, breathing, self-talk, goal setting, cognitive-behavioural therapy, or physical rehabilitation. This separation permits ability and use associations to inform construct mapping without being interpreted as intervention effects, and it prevents outcomes from embedded programmes from being attributed to imagery alone.

The outcomes charted across studies clustered in several recurring domains: competitive and state or trait anxiety and its interpretation; appraisal and emotion regulation; resilience and mental toughness; confidence, concentration, and perceived readiness; psychological fatigue and attentional recovery; anger expression; and rehabilitation-related outcomes including fear of re-injury and psychological readiness to return to sport. A subset of studies also recorded physiological or psychophysiological indices alongside psychological outcomes; studies whose only outcomes were physiological or motor-performance measures did not satisfy the second eligibility gate and were not charted. These outcomes were distributed across the four recurring, non-mutually-exclusive themes that structure the synthesis presented in the following sections.

### 3.2 Mechanisms of imagery in stress management

The proposed mechanism was interpreted as a sequence rather than as a single imagery effect. Mental simulation activates stimulus, response, and meaning representations and overlaps with perceptual and motor representations (Lang, 1979; Jeannerod, 1994). The content and function of the image may then influence appraisal, defined here as the athlete’s evaluation of competitive demands relative to perceived coping resources, and emotional response and regulation, defined as the generation, interpretation, and modulation of anxiety or affect. These processes may relate to confidence and self-efficacy, coping and resilience, attentional recovery, or injury-related fear and readiness. Figure 2 summarises this analytical pathway. It represents mechanisms proposed across the mapped literature and does not establish causal mediation.

Measured-imagery studies contributed convergent evidence on these mechanistic pathways. In a structural model of 407 elite athletes in Türkiye, imagery assessed with the Sports Imagery Inventory was associated with mental toughness and with competitive anxiety (Demir et al., 2025), and in 58 student-athletes in Pakistan healing imagery was examined as a distinct measured construct in relation to competitive anxiety and achievement goals (Ali and Kazmi, 2024). In a laboratory psychophysiological study of 30 participants, skin-conductance responding during imagery varied with sport anxiety and neuroticism (Budnik-Przybylska et al., 2023), indicating that individual characteristics condition the physiological response to imagery rather than imagery producing a uniform arousal profile. These are associational designs, and the direction of the relationships they describe cannot be established from them.

#### 3.2.1 Imagery scripts

Imagery scripts have been increasingly used in sport psychology as a stress-management strategy, and experimental evidence indicates that different script types elicit distinct psychological and physiological stress responses. Williams et al. (2017) reported that challenge-oriented imagery scripts were associated with more adaptive cardiovascular and psychological responses, whereas threat-based scripts were linked to heightened anxiety and less favourable physiological patterns, suggesting that imagery scripts may act by shaping how athletes appraise competitive demands. Complementing this, Quinton et al. (2019) found that mastery-focused imagery scripts were associated with reduced stress responses and improvements in emotional regulation and cognitive appraisal. Taken together, this evidence indicates that imagery scripts can modify athletes’ appraisal of stress, attenuate negative emotional responses, and support more adaptive coping in stressful sport situations. Extending beyond script-based imagery, Schlatter et al. (2020) examined implicit and explicit motor imagery under acute stress and reported that stress differentially affected these imagery processes, suggesting that stress may influence imagery depending on the mode of imagery being used.

#### 3.2.2 Multimodal imagery interventions

Multimodal interventions, in which imagery is combined with other psychological techniques, have been widely studied as a means of managing stress and anxiety. Such programmes commonly integrate arousal control, attention training, centring, cognitive and somatic relaxation, goal setting, self-talk, stress-inoculation training, and visuomotor behaviour rehearsal, and have been reported to target both cognitive and somatic symptoms and broader appraisal, affect, and coping processes (Rumbold et al., 2012). Within these interventions, self-talk and imagery are frequently examined together and have been associated with reductions in cognitive and somatic anxiety, while relaxation-based techniques have been associated with lower state anxiety whether used alone or with imagery.

Sport-specific evidence supports these patterns. Coelho et al. (2012) examined a multimodal imagery intervention for pre-competition anxiety and stress in tennis players and reported increased self-confidence alongside reduced anxiety and stress, suggesting benefits for self-regulation. Earlier, Mace and Carroll (1989) applied stress-inoculation training combining relaxation, imagery, and self-statements to strengthen coping capacity, and Van Raalte et al. (2019) highlighted the role of psychological preparation, including imagery, in supporting athletes’ training experiences and stress management. Collectively, multimodal approaches that integrate imagery with strategies such as arousal control and relaxation are commonly associated with reduced cognitive and somatic anxiety and improved self-confidence and psychological adjustment. Slimani et al. (2017) is retained only as a supporting source because it reported muscular-performance, hormonal, and physiological rather than eligible psychological outcomes.

Randomised evidence for imagery-centred multimodal programmes has since accumulated. In 50 male state-level table tennis players in India randomised to visuo-motor behaviour rehearsal or a control condition, the programme was associated with lower somatic anxiety, worry, and concentration disruption, although two mental-toughness subscales did not differ (Sharma and Prasad, 2023). Because imagery is co-delivered with relaxation in this paradigm, the effect is attributable to the combined package rather than to imagery alone.

Synthesising across these mechanistic sources, the evidence was most consistent in indicating that imagery influences stress-related responses through the content and appraisal of what is imagined rather than through imagery per se: challenge- and mastery-oriented representations were repeatedly associated with more adaptive affective and psychophysiological responses, whereas threat-based content was associated with heightened anxiety. Convergent signals across script-based and multimodal paradigms suggest a shared appraisal- and emotion-regulation pathway. Evidence was less consistent regarding physiological mechanisms, where indices, tasks, and sample sizes varied considerably, and where imagery was frequently combined with relaxation or breathing, making its independent physiological contribution difficult to isolate.

### 3.3 Imagery, stress management, and performance

#### 3.3.1 Psychological stress, anxiety, and performance outcomes

Athletic performance is shaped by technical, tactical, physical, and psychological factors, and psychological training (through techniques such as imagery, goal setting, and relaxation breathing) has been associated with gains in motivation, concentration, and team cohesion. Across the charted evidence, athletes ranging from Olympians to collegiate competitors were reported to use cognitive-behavioural strategies such as mental rehearsal, thought control, arousal regulation, and behavioural preparation in training and competition. Quinton et al. (2019) and Williams et al. (2017) further indicated that the effectiveness of imagery differs for cognitive versus somatic anxiety, and Williams et al. (2021) linked mastery imagery ability to more facilitative anxiety interpretations and performance under acute psychological stress. Overall, imagery and relaxation are described as strategies that help athletes manage competitive stress, sustain concentration, and maintain confidence, positioning psychological preparation as central to performance under pressure. In male rugby union players, Mellalieu et al. (2009) reported that a motivational general-arousal imagery intervention altered pre-performance symptoms and anxiety interpretation, providing sport-specific evidence that imagery can shape how athletes experience pressure before performance.

Recent intervention work has charted anxiety and confidence alongside skill outcomes. An eight-week PETTLEP programme delivered to 24 collegiate futsal players in Thailand as the sole psychological component was followed by changes in shooting skill, competitive anxiety, and self-confidence in a one-group pre-post design (Khongrassame et al., 2024), and a PETTLEP programme in 120 basketball players in China reported comparable self-reported outcomes, also without a control condition (Lu and Xu, 2023). In a pilot feasibility randomised trial, 21 female Division I volleyball players were allocated to imagery or mindfulness arms, with competitive anxiety and self-confidence charted alongside feasibility indicators (McAlister et al., 2024). A five-session imagery intervention with five female pistol shooters in the Indonesian Army likewise charted competitive anxiety as the outcome (Pratama Sudrajat et al., 2025). In a three-arm randomised trial of 30 Thai university athletes, 8 weeks of MG-A/MG-M imagery was compared with mindfulness meditation and a relaxation-brochure control (Pakulanon and Petviset, 2025). The significant reduction in cognitive state anxiety was confined to the mindfulness arm; imagery showed no significant benefit for cognitive or somatic anxiety, self-confidence, or heart-rate variability. Across these studies, small samples and the frequent absence of a control condition mean that the evidence maps how imagery is being delivered in applied settings more securely than what it achieves.

#### 3.3.2 Psychological fatigue and recovery

Loch et al. (2020) used a randomised crossover design comparing a powernap, systematic breathing, systematic breathing plus mental imagery, and a comic-reading control condition after a mentally fatiguing task. The study found no significant differences between the recovery interventions and the control condition, although subjective fatigue, recovery-stress ratings, and cognitive performance improved over time. The findings therefore do not establish an imagery-specific recovery effect.

#### 3.3.3 Cognitive-behavioural and imagery-based approaches to performance regulation

Imagery has been applied both as a component of cognitive-behavioural therapy (CBT) and as a complementary technique alongside it. Hanton et al. (2009) described how cognitive restructuring, goal setting, and imagery exercises can be used to help athletes reinterpret anxiety symptoms more facilitatively and support confidence. Olmedilla et al. (2019) examined CBT as a means of modifying cognitive patterns and behaviours, with imagery used to influence emotional states through mental representation. Ryska (1998) reported that 49 recreational tennis players (26% of the sample) used at least one cognitive-behavioural strategy during training and competition, with relaxation/imagery ranked as the most effective strategy among users; because imagery and relaxation correlated at *r* = 0.81 in that sample, they were combined into a single variable for analysis, and the resulting composite predicted lower somatic but not cognitive state anxiety. Collectively, these sources describe the use of cognitive-behavioural strategies and imagery in relation to emotional regulation, confidence, and performance-related outcomes. Similarly, Isorna-Folgar et al. (2022) examined an imagery-inclusive CBT programme with junior national-team rowers and reported improvements in motivation, concentration, cohesion, and performance-related outcomes.

Across the performance-related sources, the evidence converged on imagery being associated with anxiety interpretation, confidence, and perceived readiness, most often as part of multimodal preparation rather than as an isolated technique. Support was weaker and more heterogeneous for psychological fatigue and recovery, which rested on a small number of studies using diverse fatigue-induction and outcome measures. Differences in reported effects across this theme may reflect variation in intervention format, the competitive versus laboratory setting, the timing of assessment relative to performance, and whether outcomes were self-reported or performance-based, all of which limit direct comparison between studies.

### 3.4 Individual and sport-specific differences

#### 3.4.1 Individual characteristics of athletes

Early work suggested that individual differences were only weakly associated with the use of psychological strategies such as imagery, but subsequent evidence challenged this view. Ryska (1998) found that cognitive-behavioural strategy use among recreational tennis players was more closely related to pre-competition state anxiety than to demographic factors such as age, gender, experience, or skill level. Later studies documented clearer individual-difference effects: Williams et al. (2017) reported that female athletes showed higher anxiety, lower confidence, and distinct cardiovascular responses under acute stress, and the review by Ziv and Lidor (2013) noted gender-specific stress patterns in judo athletes undergoing weight reduction.

Westlund et al. (2012) identified a moderate association between cognitive general imagery and confidence, and adaptive coping more broadly has been linked to psychological resilience in athletes (Nicholls et al., 2008). Competitive level appears influential. Ryska (1998) described level-related differences in the prevalence, type, and perceived effectiveness of cognitive-behavioural strategies, and Erciş (2018) reported higher goal-setting and confidence scores in elite than non-elite athletes; skill-level differences in relaxation-related skills have also been reported (Kudlackova et al., 2013). Age and experience matter as well: Wang et al. (2003) noted that younger athletes engaged in shorter imagery sessions, potentially reflecting limited attentional capacity, whereas more experienced athletes sustained longer imagery practice. Stress reactivity further moderates imagery effects. Carter and Kelly (1997) found that high-reactant basketball players were more responsive to contradictory imagery, with reduced anxiety and improved confidence, whereas confidence imagery and control conditions showed limited effects. Overall, imagery use in stress management varies across gender, competitive level, resilience, confidence, and athlete maturity.

Imagery has also been positioned within models of confidence in younger athletes. In 519 adolescent athletes aged 14 to 19, imagery use assessed with the Sport Imagery Questionnaire was modelled as a mediator of the relationship between sport confidence and competitive performance (Barbosa et al., 2023), with performance indexed on a ranking scale.

#### 3.4.2 Variation across sporting disciplines

Imagery has been reported as a commonly used psychological skill across sport types, including aesthetic, power, endurance, and combat sports, and psychological-skills training in combat-sport samples has been linked to reduced competitive anxiety (Vesković et al., 2019). In figure skating, Gould et al. (1993) reported that national-level skaters used behavioural strategies, task focus, emotional regulation, and cognitive control to manage competitive pressure. Differences also emerge across sport demands: Znazen et al. (2017) found that elite power athletes scored higher than endurance athletes on skills such as goal setting, fear control, imagery, and competition planning. The review by Ziv and Lidor (2013) reported greater imagery engagement among judo medallists than non-medallists, though it remained unclear whether this reflected performance, stress management, or other contextual factors, and also noted elevated tension, anger, fatigue, and confusion among athletes in weight-loss programmes. Collectively, these findings indicate that imagery use and stress responses vary with sport-specific demands and competitive contexts rather than following a uniform pattern. In Olympic wrestling, Gould et al. (1992) described mental rehearsal as part of athletes’ broader mental-preparation and coping repertoire, illustrating how imagery-related strategies are adapted to high-pressure elite sport contexts.

Taken together, the sources in this theme indicate that engagement with imagery and its associated effects are not uniform but vary with individual characteristics, including gender, competitive level, experience, and stress reactivity, and with sport-specific demands across aesthetic, combat, power, and endurance disciplines. The evidence was consistent in demonstrating that variation exists, but less consistent in specifying its direction or magnitude, in part because studies differed in the moderators examined and rarely tested the same characteristic across comparable samples. This heterogeneity cautions against a single, uniform model of imagery-based stress management and supports individualised, context-sensitive application.

### 3.5 Imagery in injury prevention and rehabilitation

Several sources examined the relationship between psychological stress and sport injury and the role of psychological factors during injury and recovery. Hardy (1992) described multiple stress sources reported by athletes, including fear of failure, social-evaluation concerns, perceived inadequate preparation, and loss of control, which have been integrated into theoretical accounts linking stress to injury risk, most notably the stress–injury model of Williams and Andersen (1998), which emphasises cognitive appraisal, attentional focus, and physiological responses. Across these sources, techniques used for performance enhancement, namely goal setting, imagery, self-talk, and relaxation, were also applied in injury-related contexts.

#### 3.5.1 Injury prevention

Contextual evidence on the stress–injury relationship indicates that higher stress levels are associated with an increased likelihood of sport-related injury, particularly under high-stress conditions (van Winden et al., 2021), providing a rationale for applying psychological strategies in injury prevention. Within psychological stress-management and psychological-skills intervention work, targeted programmes have been linked with reduced injury incidence, including in youth sport. Olmedilla-Zafra et al. (2017) reported that a psychological stress-management programme was associated with reduced injury incidence among young football players. Linking psychological profiles to outcomes, Sajedi et al. (2020) used the OMSAT-3 and reported inverse associations between injury risk and overall, basic, and psychosomatic psychological-skill scores; this source is drawn on for context rather than charted, because imagery is subsumed within a broader skill category and is not reported separately. Collectively, these sources describe associations between psychological skills, stress-management capacity, and injury-related outcomes.

#### 3.5.2 Recovery from sport injury

Several sources examined psychological processes during rehabilitation. In a systematic review, Schwab Reese et al. (2012) described how injured athletes frequently experience psychological challenges during recovery despite faster physical healing, and how imagery and relaxation were used to support task-focused attention and emotional regulation, for example by having athletes visualise rehabilitation progress and return-to-play scenarios, with reported reductions in re-injury anxiety and improved coping. Johnson (2000) examined guided imagery in 58 injured athletes and reported improved overall mood, using techniques that involved visualising healing processes and functional movement. Case-based evidence from Davis and Sime (2005) described imagery combined with EEG biofeedback during rehabilitation, with improvements in confidence and subsequent performance; however, these gains diminished when imagery practice was discontinued. A more recent single-case report (Olds, 2025) applied graded exposure to fear-eliciting images as a first-person motor imagery task alongside usual postoperative physiotherapy in an athlete with recurrent shoulder instability; kinesiophobia measured on the TSK-11 fell from 33/44 to 24/44, a change exceeding the reported minimal detectable change, although imagery was one component of a concurrent rehabilitation programme and the change cannot be attributed to imagery alone. One further pilot study of imagery during rehabilitation following anterior cruciate ligament surgery (Kasmi et al., 2026) was retained as supporting evidence rather than charted, because its reported outcomes were confined to physical function and imagery ability, with no eligible stress-related psychological outcome.

Within the injury theme, the evidence was reasonably consistent in linking psychological-skills provision incorporating imagery to lower injury incidence in prevention contexts and to improved mood, coping, and reduced re-injury anxiety in rehabilitation. However, this strand rested on relatively few sources, imagery was almost always delivered alongside relaxation or broader cognitive-behavioural techniques, and outcome definitions ranged from injury counts to psychological recovery indices. Reported benefits also appeared contingent on continued practice. These features mean that, while the direction of evidence is encouraging, the specific role of imagery within injury-related intervention packages remains difficult to isolate from the charted sources.

## 4 Discussion

This scoping review charted 39 core empirical studies on the use of mental imagery for stress management in athletes, drawn from 1,004 de-duplicated records screened across two search waves, and drew additionally on a set of supporting review, applied, and adjacent empirical sources in which imagery formed part of a broader psychological-skills approach. The supporting sources were used to characterise how imagery is used in practice, while the charted synthesis was restricted to studies in which imagery was an operationally distinct construct examined against a psychological outcome. Read as a whole, the mapped literature suggests that imagery is best understood not as a single technique with a fixed effect, but as a flexible psychological process that is embedded within broader skill sets and adapted to the demands of particular athletes and contexts. Three cross-cutting patterns emerged from the mapping and organise the discussion that follows: imagery operates through appraisal- and emotion-related mechanisms; it is rarely delivered in isolation; and its use and reported effects are strongly context-dependent. A fourth observation, which the updated search brought into focus, cuts across all three: several of the most recent and methodologically explicit studies reported null or inconclusive results, and these are discussed alongside the positive findings rather than set aside.

### 4.1 Imagery mechanisms and stress regulation in sport

The distinction between what is imagined and how imagery functions has been central to the field since Paivio (1985) separated the cognitive and motivational functions of imagery, and it is embedded in the instruments most widely used to assess imagery in athletes (Hall et al., 1998). Applying the four-dimensional framework shows that the mapped mechanisms arise from different forms of evidence. Imagery-content studies compared challenge, threat, and mastery representations and linked these contents to appraisal and affective responses (Williams et al., 2017; Quinton et al., 2019). Imagery-ability studies examined whether capacity to generate or control images was associated with anxiety interpretation, confidence, or stress responses, while imagery-use studies examined when and why athletes deliberately imaged. Intervention-format studies tested standalone or embedded delivery. These findings are complementary, but they answer different questions and should not be pooled conceptually as evidence of one uniform imagery effect.

Bio-informational theory provides a mechanism for content effects because stimulus, response, and meaning propositions can alter the represented emotional situation (Lang, 1979). Functional equivalence and PETTLEP explain how perceptual and action representations may be strengthened by matching imagery to performance conditions (Jeannerod, 1994; Holmes and Collins, 2001). The revised applied model of deliberate imagery use adds the athlete’s purpose, situation, and meaning, which helps explain why identical imagery formats may produce different psychological responses (Cumming and Williams, 2013). Together, these accounts support an appraisal and emotion-regulation pathway, but the current evidence does not establish that this pathway mediates intervention effects.

### 4.2 Imagery as an embedded, multimodal strategy

A second cross-cutting pattern concerns intervention format. Imagery was frequently embedded within programmes that also included relaxation, self-talk, arousal control, goal setting, motivational interviewing, or physical rehabilitation (Rumbold et al., 2012; Coelho et al., 2012; Mace and Carroll, 1989; Norouzi et al., 2025). This reflects applied practice but limits attribution. The mapped literature can show that imagery is commonly used within multicomponent provision and that favourable outcomes were reported after some packages. It cannot establish that embedded delivery is superior to standalone imagery or that imagery was the active component.

### 4.3 Context-dependence: individual and sport-specific variation

A third consistent finding is that imagery use and its reported effects are context-dependent rather than uniform. Individual characteristics, including gender, competitive level, experience and maturity, and stress reactivity, were repeatedly associated with differences in how athletes engage with imagery and related strategies (Williams et al., 2017; Ryska, 1998; Wang et al., 2003; Carter and Kelly, 1997). Sport-specific demands added a further layer, with imagery adapted across aesthetic, power, endurance, and combat sports to meet different cognitive and emotional requirements (Znazen et al., 2017; Vesković et al., 2019). Taken together, this variation argues against a one-size-fits-all model of imagery-based stress management and supports the case for individualised, sport-tailored approaches. It also helps explain the heterogeneity of reported effects across the literature, since studies differ substantially in the populations and contexts sampled.

Two recent meta-analyses bear on the same question from a pooled-estimate perspective. Li et al. (2026) synthesised trials of differing psychological interventions on competitive-state anxiety in athletes, and Zhang et al. (2025) examined the dosage and delivery parameters of imagery practice in relation to athletes’ mental skills. Both are drawn on for context rather than charted, because a meta-analysis pools primary studies rather than contributing a study-level record to the evidence map. Read cautiously, they are consistent with the context-dependence described above: pooled estimates in this literature rest on heterogeneous protocols, and the parameters that appear to matter, notably session frequency and total exposure, are precisely those the charted primary studies most often leave unspecified. Their pooled estimates should not be read as establishing an isolated imagery effect, since the underlying trials rarely separate imagery from co-delivered components.

### 4.4 Imagery in performance, fatigue, and injury contexts

Beyond acute competitive stress, the mapped literature extends imagery to performance readiness, psychological fatigue, and the injury cycle. Psychological training incorporating imagery was associated with confidence, concentration, and perceived readiness. However, Loch et al. (2020) found no between-condition advantage for systematic breathing plus imagery over a comic-reading control following mental fatigue. In injury contexts, imagery appeared in prevention and rehabilitation programmes, but formats and co-delivered components varied widely. These studies map where imagery has been applied; they do not establish a uniform recovery or injury-related benefit.

### 4.5 Null and inconclusive findings

Mapping the updated evidence brings into view a set of findings that qualify the generally positive picture conveyed by the earlier literature, and reporting them is necessary if the map is to represent the field accurately. In a randomised trial of motor imagery added to rehabilitation in professional footballers with Grade II ankle sprain, fear of re-injury did not differ between the imagery and placebo groups, although some balance outcomes did (Plakoutsis et al., 2024). In a feasibility study of an integrated motor-imagery and physical-training intervention following knee injury, the pre-specified progression criteria were not met, so the intervention was not judged ready for a definitive trial (Cederström et al., 2024). In a cross-sectional model of imagery use and psychological resilience in student athletes, sport confidence did not mediate the relationship, contrary to the hypothesised pathway, even though several imagery functions correlated with resilience (D'Agostino and Munroe-Chandler, 2025). These results sit alongside a mediation study in a different population and with a different mediator, in which general self-efficacy did carry part of the association between imagery and perceived stress (Di Corrado et al., 2025). The two mediation studies used different instruments, different mediators, and different samples, and their divergence should be read as evidence that the mediating pathway is not yet established rather than as a contradiction to be resolved in favour of either result. A cross-sectional study of elite athletes reported that imagery ability was associated with body awareness but showed essentially null associations across psychological-skill subscales, the only significant relationship being a negative one involving the worry-related subscale (Emirzeoglu et al., 2024). This pattern is informative rather than anomalous, because it indicates that imagery ability need not translate into the psychological-skill profile that applied accounts typically assume. A three-arm randomised trial of functional imagery training and motor imagery in elite handball players reported that emotion dysregulation, anxiety, and perceived stress declined across all three groups, including the control condition, so the advantage attributable to imagery was confined to reaction-time performance (Norouzi et al., 2025). In a within-subject experiment manipulating crowd noise during penalty taking, performance declined under negative noise, but no psychological skill, imagery included, moderated that effect (Oldfield et al., 2026). An eight-week three-arm trial likewise found no significant imagery-group benefit for cognitive or somatic anxiety, self-confidence, or heart-rate variability; the significant cognitive-anxiety reduction was confined to the mindfulness group (Pakulanon and Petviset, 2025). Taken together, these findings indicate that the relationship between imagery and stress-related outcomes is neither uniform nor uniformly positive, and that rehabilitation contexts in particular have produced negative results on precisely the psychological outcomes that imagery is often assumed to address.

A further supporting source points in the same direction on physiological indices. Lee et al. (2023) reported autonomic nervous system responses following psychological-skills training that included imagery, in five athletes without a control condition; this source is drawn on for context rather than charted, because imagery formed one component of a multicomponent programme and its contribution cannot be separated. The physiological strand of this literature shares the limitation that characterises the psychological strand: the outcomes most often invoked to explain how imagery regulates stress are assessed in designs too small or too confounded to attribute change to imagery itself.

### 4.6 Limitations

Several limitations should be considered. No formal risk-of-bias or methodological quality appraisal was undertaken because the objective was to map the extent, range, and nature of the evidence rather than estimate efficacy (Arksey and O’Malley, 2005; Levac et al., 2010). The review therefore does not support causal or comparative-effectiveness claims. ZL and WY independently screened all 1,004 title and abstract records. At the full-text stage, each reviewer applied the operational gates to the reports retained through their own screen; WY retained 71 reports, and five discrepant core-study classifications were resolved through discussion, with two included in the final core empirical set. Full-text data charting, however, was primarily conducted by ZL and was not independently duplicated for every charted study, so extraction or classification error remains possible. The evidence was heterogeneous in imagery construct, delivery, and outcome measurement, and imagery was often embedded within multicomponent programmes. The search was limited to four databases, non-English reports were excluded during screening, and 18 reports could not be retrieved. Relevant grey literature and non-English evidence may therefore have been missed. Finally, no protocol was prospectively registered, although the research questions, eligibility criteria, and charting framework were specified in advance.

A recurring implication across these patterns concerns the kind of claim the present review can support. Because it mapped rather than appraised the evidence, and because imagery was typically embedded within multimodal programmes, this review cannot establish the efficacy of imagery as an isolated intervention or compare effect magnitudes across approaches. It can instead clarify the structure of the field: the extent and recency of the evidence, the range of designs, populations, and outcomes, the recurring mechanisms proposed, and the boundaries within which imagery has been applied. This distinction defines the scope of the review and informs whether and where more constrained syntheses are now warranted.

### 4.7 Implications for practice

For practitioners, the map shows that imagery is commonly delivered within broader psychological-skills programmes and adapted to the athlete, task, and context. This pattern describes prevailing practice; it does not demonstrate that embedded delivery is more effective than standalone imagery. Until component-controlled studies isolate imagery’s contribution, imagery should be used provisionally, individualised according to ability, content, purpose, and delivery format, and monitored against clearly defined psychological outcomes. Uptake among sport practitioners may also lag behind the research literature: in a survey of 102 strength and conditioning practitioners, imagery was the least frequently used of 11 psychological skills and strategies, with goal setting the most frequently used (Radcliffe et al., 2013), which suggests that the practical dissemination of imagery techniques warrants attention alongside their evaluation.

### 4.8 Future research

Future research should report the four imagery dimensions separately: ability, content and function, use, and intervention format. Component-controlled or dismantling designs are needed to isolate imagery from co-delivered techniques. Studies should specify the image content, intended function, frequency, timing, setting, delivery procedure, and the instrument used to assess ability or use. Adequately powered longitudinal and cross-seasonal designs should test proposed appraisal and emotion-regulation pathways and examine variation across athlete groups, sports, competitive levels, and under-represented cultural contexts. A further extension concerns sport populations outside the athlete role. Competitive stress is also borne by match officials, whose coping repertoires have been described in their own right (Hajji et al., 2026); whether imagery-based stress management transfers to these groups falls outside the population scope of the present review and remains an open question.

## 5 Conclusion

This scoping review charted 39 core empirical studies from 1,004 de-duplicated records and distinguished imagery ability, imagery content and function, imagery use, and intervention format. The mapped literature links imagery to appraisal, emotion regulation, confidence, coping, fatigue, and injury-related outcomes across varied sport contexts, but the forms of evidence answer different questions. Ability and use associations do not demonstrate intervention efficacy, and outcomes from multicomponent programmes cannot usually be attributed to imagery alone. The most important next steps are clearer construct reporting, component-controlled designs, longitudinal tests of proposed mechanisms, and broader athlete populations.

## Data Availability

The original contributions presented in the study are included in the article/Supplementary material, further inquiries can be directed to the corresponding author.

## Supplementary material

Supplementary Table S1 Search strategy: concepts, keyword combinations and Boolean logic per database, database-specific record yields for both search waves, and the dated deduplication audit.

Supplementary Table S2 Study-level charting record for all retained sources, including source role, design, sample and sport context, imagery dimensions coded, outcomes and reporting caveats.
