# Supplementary material for: Mapping the use of mental imagery for stress management in athletes: a scoping review
Source: Front Psychol. 2026 Sep 23;17:1931753. doi: 10.3389/fpsyg.2026.1931753 (PMC13642190; doi:10.3389/fpsyg.2026.1931753)
Supplement: Supplementary file 1 [file Data_sheet_1.pdf]

Supplementary Material

Supplementary Table S1. Search strategy: concepts, keywords, Boolean logic, database yields, and deduplication audit for both search waves.

Part A. Search concepts, keywords, and Boolean logic

Concept block (P/C/C)	Keywords (combined within block using OR)
Concept 1: Mental imagery	"mental imagery" OR "imagery" OR "visualisation" OR "visualization" OR "mental rehearsal"
Concept 2: Athletes and sport participation	"athlete" OR "athletes" OR "sport" OR "sports" OR "player" OR "competitor"
Concept 3: Stress management and related outcomes	"stress" OR "stress management" OR "anxiety" OR "coping" OR "performance pressure" OR "injury prevention" OR "rehabilitation"
Boolean combination across blocks	Concept 1 AND Concept 2 AND Concept 3

Part B. Records identified by database and search wave

Database (platform)	Concept structure applied	Limits at search stage	Wave 1: initial search (January 2024)	Wave 2: updated search (27 July 2026)	Total identified
Web of Science	C1 AND C2 AND C3	Initial: none; update: Jan 2023-Jul 2026; no language limit	101	254	355
Scopus	C1 AND C2 AND C3	Initial: none; update: Jan 2023-Jul 2026; no language limit	24	347	371
PubMed	C1 AND C2 AND C3	Initial: none; update: Jan 2023-Jul 2026; no language limit	416	121	537
EBSCOhost	C1 AND C2 AND C3	Initial: none; update: Jan 2023-Jul 2026; no language limit	22	219	241
Total identified			563	941	1,504

Part C. Deduplication audit

Stage	Wave 1	Wave 2	Combined
Records identified	563	941	1,504
Duplicate records removed	38	462	500
Of which duplicated within the EBSCOhost export	0	175	175
Unique records taken forward to title and abstract screening	525	479	1,004

Note. Part A presents the search concepts and Boolean structure applied across the four databases or database platforms. Within each concept block, keywords were combined using OR, and the three blocks were combined using AND. Database-specific syntax was standardised for presentation rather than reproduced verbatim. The initial search was conducted in January 2024 with no lower publication-year limit. The updated search was conducted on 27 July 2026 with coverage from January 2023 to July 2026, so the update overlapped the end of the original window. No language limit was applied at the search stage; non-English records were excluded during screening. Duplicate detection was performed within and across waves. The 500 duplicate records removed comprised 38 from the initial wave and 462 from the updated wave, including 175 duplicates within the EBSCOhost export. ZL and WY independently screened all 1,004 de-duplicated records at title and abstract stage. EBSCOhost refers to the EBSCO search platform.

Supplementary Table S2. Evidence charting of the 39 core empirical studies

Note. Studies are numbered 1 to 39 in the same order as Table 1 in the main article. Ordering is by search wave and then year of publication. Search wave indicates whether the record was identified in the original or updated search. Reporting caveats, including null findings and outcomes that could not be attributed specifically to imagery, are given in the Main stress-related relevance column.

Ref	Author (year)	Sample / sport	Design / source type	Imagery approach	Outcome(s) measured	Theme(s)	Delivery	Main stress-related relevance	Search wave
1	Mace & Carroll (1989)	Gymnasts	Experimental / intervention (SIT)	Imagery within stress-inoculation training	Self-report/observer stress / HR / performance	Mechanisms (multimodal); Performance	Embedded / multimodal	Imagery-based SIT strengthened coping and reduced stress indices under performance demands	Original
2	Gould, Eklund & Jackson (1992)	Olympic wrestlers	Qualitative / interview	Mental preparation incl. mental rehearsal/visualization	Mental preparation / precompetitive cognition / affect	Individual/sport-specific; Performance	Embedded / multimodal	Mental rehearsal/visualization formed part of Olympic wrestlers' broader mental-preparation and coping repertoire	Original
3	Gould, Finch & Jackson (1993)	National-champion figure skaters	Qualitative / interview	Imagery among emotion-regulation & cognitive-control strategies	Coping / emotion regulation / performance under stress	Individual/sport-specific; Performance	Embedded / multimodal	Skaters used imagery within a repertoire of strategies to regulate emotion under competitive stress	Original
4	Carter & Kelly (1997)	Intramural basketball players	Experimental	Confidence vs contradictory imagery	Anxiety / confidence (by reactance)	Individual (reactance); Mechanisms	Imagery measured as a construct (non-intervention)	Response to imagery varied with psychological reactance	Original
5	Ryska (1998)	Recreational tennis players	Cross-sectional survey	Mental imagery (four tennis-specific referents); merged with relaxation for analysis owing to collinearity (r = .81)	Precompetitive anxiety / strategy use	Individual; Performance	Embedded / multimodal	Strategy use tied to pre-competition state anxiety; imagery/relaxation rated most effective among users	Original
6	Johnson (2000)	Long-term-injured competitive athletes	Intervention (rehab)	Guided imagery	Mood / psychological recovery	Injury (recovery)	Standalone imagery	Guided imagery associated with improved mood during rehabilitation	Original
7	Wang, Callahan & Goldfine (2003)	Athletes (choking under pressure)	Intervention / applied	Psychological intervention incl. imagery	Choking / pressure / imagery practice	Performance; Individual (age)	Embedded / multimodal	Imagery-inclusive intervention addressed choking and pressure; imagery dose varied by age	Original
8	Davis & Sime (2005)	Injured athlete(s) (case-based)	Case study	Imagery + EEG biofeedback	Confidence / performance post-recovery	Injury (recovery)	Embedded / multimodal	Imagery with biofeedback aided confidence/performance in recovery; gains diminished when discontinued	Original
9	Mellalieu, Hanton & Thomas (2009)	Male rugby union players	Single-case intervention	Motivational general-arousal (MG-A) imagery	Pre-performance symptoms / anxiety interpretation	Performance	Standalone imagery	MG-A imagery altered pre-performance symptoms and anxiety interpretation	Original
10	Coelho et al. (2012)	Elite tennis players	Intervention (pre–post)	Multimodal imagery intervention	Pre-comp anxiety↓ / stress↓ / confidence↑	Mechanisms (multimodal); Performance	Embedded / multimodal	Reduced pre-competition anxiety and stress; increased self-confidence	Original
11	Westlund, Pope & Tobin (2012)	Athletes	Cross-sectional / correlational	Cognitive general (CG) imagery	Confidence (CG imagery association)	Individual (confidence); Mechanisms	Imagery measured as a construct (non-intervention)	CG imagery associated with confidence	Original
12	Olmedilla-Zafra et al. (2017)	Young football players	Intervention (prevention)	Imagery within a stress-management programme	Injury incidence	Injury (prevention)	Embedded / multimodal	Stress-management programme incorporating imagery linked to reduced injury incidence	Original
13	Williams et al. (2017)	Lab acute-stress sample (mixed sex)	Experimental (imagery scripts)	Challenge vs threat imagery scripts	Anxiety / cardiovascular (HR) / appraisal	Mechanisms; Individual (gender)	Standalone imagery	Script content shaped anxiety, appraisal and cardiovascular stress responses; sex differences observed	Original
14	Olmedilla et al. (2019)	Youth soccer players	Intervention (prevention)	Imagery within a psychological programme	Stress control / injury incidence	Injury (prevention); Performance	Embedded / multimodal	Programme incorporating imagery linked to stress control and injury incidence	Original
15	Quinton et al. (2019)	Athletes (acute stress task)	Experimental (imagery scripts)	Mastery imagery (ability/scripts)	Stress responses / emotion regulation / appraisal	Mechanisms	Imagery measured as a construct (non-intervention)	Mastery imagery linked to more adaptive stress responses and emotion regulation	Original
16	Vesković et al. (2019)	Top karate athletes	Intervention (PST)	Imagery within psychological-skills training	Competitive anxiety	Performance/anxiety; Sport-specific (combat)	Embedded / multimodal	PST incorporating imagery linked to reduced competitive anxiety	Original
17	Loch et al. (2020)	Mentally fatigued participants	Experimental	Imagery + breathing as recovery strategy	Psychological fatigue / attention / cognitive performance	Performance (fatigue & recovery)	Embedded / multimodal	No significant between-condition differences were reported; the study does not establish an imagery-specific recovery benefit.	Original
18	Schlatter et al. (2020)	Athletes (acute stress; pilot)	Experimental (pilot)	Implicit vs explicit motor imagery	Implicit vs explicit motor imagery under stress	Mechanisms (motor imagery); Sport-specific	Imagery measured as a construct (non-intervention)	Acute stress differentially affected implicit vs explicit motor imagery	Original
19	Williams et al. (2021)	Athletes (psychological stress)	Experimental	Mastery imagery ability	Positive anxiety / performance / confidence	Mechanisms; Performance; Individual	Imagery measured as a construct (non-intervention)	Mastery imagery ability linked to facilitative anxiety interpretation and performance under stress	Original
20	Isorna-Folgar et al. (2022)	Junior national-team rowers	Intervention (CBT)	Imagery within a CBT programme	Motivation / concentration / cohesion / performance	Performance	Embedded / multimodal	Imagery-inclusive CBT linked to motivation, concentration, cohesion and performance	Original
21	Barbosa et al. (2023)	519 adolescent athletes (14-19 yr)	Cross-sectional / SEM	Sport Imagery Questionnaire (SIQ) in a mediation model	Sport confidence; competitive performance	Mechanisms; Performance	Imagery measured as a construct (non-intervention)	Imagery tested as a mediator of the confidence-performance relationship. Several reported betas are negative in a direction that is hard to reconcile with the stated aims; verify sign conventions before citing coefficients.	Update 2026
22	Budnik-Przybylska et al. (2023)	30 participants, 14-42 yr, laboratory	Laboratory correlational psychophysiology	Imagery Skills Questionnaire; Sport Anxiety Scale; skin conductance level (SCL/EDA)	Physiological arousal (SCL) during imagery; sport anxiety; neuroticism as moderators	Mechanisms; Individual/sport-specific	Imagery measured as a construct (non-intervention)	Maps how individual characteristics condition physiological responding during imagery; not an intervention study.	Update 2026
23	Lu & Xu (2023)	120 basketball players, China	Intervention (pre-post, no control)	PETTLEP imagery	Self-report psychological outcomes	Mechanisms; Performance	Standalone imagery	No control group; self-report only, so time, training and expectancy effects cannot be excluded.	Update 2026
24	Sharma & Prasad (2023)	50 male state-level table tennis players, India (25 VMBR / 25 control), 17-23 yr	Randomised controlled trial	Visuo-motor behaviour rehearsal (imagery + relaxation)	Anxiety dimensions (somatic, worry, concentration disruption); mental toughness (confidence and consistency subscales non-significant)	Mechanisms; Multimodal	Embedded / multimodal	Imagery-centred multimodal programme; imagery is the organising component but is co-delivered with relaxation, so it is not an imagery-only effect.	Update 2026

Ref	Author (year)	Sample / sport	Design / source type	Imagery approach	Outcome(s) measured	Theme(s)	Delivery	Main stress-related relevance	Search wave
25	Ali & Kazmi (2024)	58 student-athletes, Pakistan	Cross-sectional / mediation	Healing imagery (measured)	Competitive sport anxiety; achievement goals	Mechanisms	Imagery measured as a construct (non-intervention)	Healing imagery examined as a distinct measured construct in relation to competitive anxiety; cross-sectional, associational only.	Update 2026
26	Cederström et al. (2024)	42 people rehabilitating traumatic knee injury (MOTIFS vs care-as-usual, 12 weeks)	Randomised controlled trial (adaptive; interim analysis)	Integrated motor imagery plus physical training	Psychological readiness to return to sport	Injury (recovery)	Embedded / multimodal	Pre-specified progression criteria were not met: a negative or inconclusive result that should be reported as such.	Update 2026
27	Emirzeoglu et al. (2024)	53 elite athletes	Cross-sectional / correlational	MIQ-3 internal, external and kinaesthetic imagery ability	Body awareness; athletic psychological skills subscales (largely null; one negative association involving worry)	Mechanisms (null findings); Individual/sport-specific	Imagery measured as a construct (non-intervention)	Imagery ability treated as a distinct measured construct and related statistically to psychological outcomes.	Update 2026
28	Khongrassame et al. (2024)	24 collegiate futsal players, Thailand	Intervention (8-week PETTLEP), one-group pre-post, no control group	PETTLEP imagery, 8 weeks	Shooting skill; competitive anxiety; self-confidence	Mechanisms; Performance	Standalone imagery	PETTLEP imagery delivered as the sole psychological component, with anxiety and confidence charted alongside a skill outcome.	Update 2026
29	McAlister et al. (2024)	21 female Division I volleyball players, USA	Pilot feasibility RCT	Imagery vs mindfulness (separate arms)	CSAI-2R anxiety and self-confidence; feasibility	Mechanisms; Performance	Standalone imagery	Pilot comparison of imagery with mindfulness; reported direction of the self-confidence change is inconsistent between text and table, so the magnitude is not citable.	Update 2026
30	Plakoutsis et al. (2024)	58 professional footballers with Grade II ankle sprain (29 motor imagery / 29 placebo), Greece	RCT	Motor imagery added to rehabilitation	Static and dynamic balance; heart rate; fear of re-injury (no between-group difference on fear of re-injury)	Injury (recovery)	Embedded / multimodal	Fear of re-injury did not differ between groups: an explicit negative finding that constrains claims about imagery in rehabilitation.	Update 2026
31	D'Agostino & Munroe-Chandler (2025)	243 student-athletes, Canada and USA	Cross-sectional / SEM	SIQ five imagery functions	Psychological resilience (CD-RISC); sport confidence as mediator	Mechanisms	Imagery measured as a construct (non-intervention)	Resilience correlated with several imagery functions, most strongly MG-M (r = .481); sport confidence did not mediate. Model fit indices are internally inconsistent, so only the bivariate pattern and the null mediation are citable.	Update 2026
32	Demir et al. (2025)	407 elite athletes (143 female / 264 male), Turkiye, 19-26 yr	Cross-sectional / SEM	Sports Imagery Inventory (SII); Mental Toughness Scale; ESS anxiety subscale	Mental toughness; anxiety (SEM paths; athletic performance not measured)	Mechanisms; Performance	Imagery measured as a construct (non-intervention)	Imagery and anxiety both entered the structural model; associations only, no directional claim.	Update 2026
33	Di Corrado et al. (2025)	110 karate, taekwondo and judo athletes, Italy	Cross-sectional / mediation	VVIQ, VMIQ-2 and a mental image transformation task	Perceived stress; general self-efficacy as mediator	Mechanisms	Imagery measured as a construct (non-intervention)	Imagery measured with three dedicated instruments and analysed against a stress outcome. Effect estimates are not citable (a standardised indirect effect exceeds its theoretical bound); the design is cross-sectional.	Update 2026
34	Norouzi et al. (2025)	n = 36 elite female handball players, post-COVID-19 motor function reduction	Randomised controlled trial (three arms), pre-test / post-test / 12-week follow-up	Functional imagery training (FIT, imagery + motivational interviewing) vs motor imagery; 12 sessions over 4 weeks, 3 × 30 min per week	DERS (emotion dysregulation); BAI (anxiety); PSS (perceived stress); NSMT reaction time (DERS, BAI and PSS declined in all three groups; the imagery advantage was confined to reaction time)	Mechanisms; Performance/fatigue	Standalone imagery and imagery plus motivational interviewing	Emotion dysregulation, anxiety and perceived stress declined in all three groups including the control condition, so the advantage attributable to imagery was confined to reaction-time performance. Recorded as a qualifying rather than confirmatory result.	Update 2026
35	Olds (2025)	n = 1 male athlete aged 23, recurrent shoulder instability after repeated stabilisation surgery, New Zealand	Single-case report	Graded exposure to fear-eliciting images used as a first-person motor imagery task, adjunct to usual physiotherapy	Kinesiophobia (TSK-11); per-image fear rating; shoulder strength and range of motion	Injury (recovery); mechanisms	Imagery adjunct to usual physiotherapy	Fear-eliciting images were used directly as the imagery task, targeting kinesiophobia during rehabilitation; as a single case without a control condition, the observed change cannot be attributed to imagery alone.	Update 2026
36	Pratama Sudrajat et al. (2025)	5 female pistol shooters, Indonesian Army	Intervention (one-group pre-post)	Mental imagery, 5 sessions	CSAI-2R competitive anxiety	Mechanisms; Performance	Standalone imagery	Imagery as the sole intervention with a competitive-anxiety outcome, but n = 5 with no control group; the reported test statistic and the N-Gain signs are internally inconsistent.	Update 2026
37	Di Corrado et al. (2026)	120 male adolescent rugby players, Italy (62 experimental / 58 control), mean age 16.9 yr	Randomised controlled trial	Personalised mental imagery training	Anger expression; resilience	Mechanisms; Individual/sport-specific	Standalone imagery	Personalised imagery targeting emotion regulation; scale-direction reporting requires care before any coefficient is cited.	Update 2026
38	Oldfield et al. (2026)	24 footballers	Within-subject experimental	Imagery measured after each crowd-noise condition	Imagery, self-talk, relaxation and emotional control; penalty performance (no psychological skill, including imagery, moderated the crowd-noise effect)	Mechanisms; Performance	Imagery measured as a construct (non-intervention)	Imagery use measured under positive, negative, stressful and no-noise conditions, linking psychological-skill use to a pressure manipulation.	Update 2026
39	Pakulanon & Petviset (2025)	30 university athletes, Thailand (badminton, athletics, football and swimming), 18-25 yr	Three-arm randomised controlled trial (n = 10 per arm), 8 weeks	MG-A/MG-M guided imagery vs mindfulness meditation vs relaxation-brochure control	CSAI-2R cognitive and somatic anxiety and self-confidence; HRV (supporting physiological outcome)	Performance/anxiety (null findings)	Imagery-centred group sessions, 30 min, 3 times/week	The significant cognitive-anxiety reduction was confined to the mindfulness arm; imagery showed no significant benefit for cognitive or somatic anxiety, self-confidence, or HRV. Small arms (n = 10) and unmeasured imagery ability limit inference.	Update 2026
